# Increase in reptile-associated human salmonellosis and shift toward adulthood in the age groups at risk, the Netherlands, 1985 to 2014

**DOI:** 10.2807/1560-7917.ES.2016.21.34.30324

**Published:** 2016-08-25

**Authors:** Lapo Mughini-Gras, Max Heck, Wilfrid van Pelt

**Affiliations:** 1 National Institute for Public Health and the Environment (RIVM), Centre for Infectious Disease Control, Bilthoven, the Netherlands

**Keywords:** *Salmonella*, salmonellosis, laboratory surveillance, zoonotic infections

## Abstract

While the contribution of the main food-related sources to human salmonellosis is well documented, knowledge on the contribution of reptiles is limited. We quantified and examined trends in reptile-associated salmonellosis in the Netherlands during a 30-year period, from 1985 to 2014. Using source attribution analysis, we estimated that 2% (95% confidence interval: 1.3–2.8) of all sporadic/domestic human salmonellosis cases reported in the Netherlands during the study period (n = 63,718) originated from reptiles. The estimated annual fraction of reptile-associated salmonellosis cases ranged from a minimum of 0.3% (corresponding to 11 cases) in 1988 to a maximum of 9.3% (93 cases) in 2013. There was a significant increasing trend in reptile-associated salmonellosis cases (+ 19% annually) and a shift towards adulthood in the age groups at highest risk, while the proportion of reptile-associated salmonellosis cases among those up to four years-old decreased by 4% annually and the proportion of cases aged 45 to 74 years increased by 20% annually. We hypothesise that these findings may be the effect of the increased number and variety of reptiles that are kept as pets, calling for further attention to the issue of safe reptile–human interaction and for reinforced hygiene recommendations for reptile owners.

## Introduction

*Salmonella* is a natural inhabitant of the reptile gut microflora, detected in ca 50% of reptile pets [1]. As pet reptiles have become increasingly popular, so have reptile-associated *Salmonella* infections in humans [2]. Most *Salmonella* isolates from reptiles belong to the *Salmonella enterica* subspecies II (*salamae*), IIIa (*arizonae*), IIIb (*diarizonae*), VI (*houtenae*), and a few to *S. bongori* (formerly subspecies V) and VI (*indica*). However, also the subspecies I (*enterica*), mainly associated with warm-blooded organisms, is often found in reptiles [3], as it can be present in the reptiles’ meals (e.g. rodents, birds or raw vegetables). Accordingly, exposure to reptiles is associated with a four- and twofold increased risk for infection with typical and atypical reptile-associated *Salmonella*, respectively [2]. Moreover, reptile-associated salmonellosis mainly affects young children and results in a higher incidence of hospitalisation and invasive disease than other *Salmonella* infections [2].

The contribution of the main food-related sources to human salmonellosis is well documented [4-7]. In contrast, the knowledge on the contribution of reptiles is limited [3]. In order to address this knowledge gap, we quantified and examined trends in reptile-associated salmonellosis in the Netherlands during a 30-year period from 1985 to 2014.

## Methods

We performed source attribution of human salmonellosis cases using the modified Dutch model, which has been presented in detail previously [4,5,8]. Briefly, the model infers probabilistically the most likely sources of human cases by comparing their *Salmonella* subtype distribution with that of the sources, weighted by the *Salmonella* prevalence in these sources and the human exposure to them, i.e. the per capita food consumption and likelihood of consuming raw/undercooked food or the per capita ownership of reptiles in the general population. Model parameters are summarised in Table 1.

**Table 1:** Parameters of the modified Dutch model for source attribution

Parameter	Description/estimation	Reference
*λ_ij_*	Estimated number of human infections caused by subtype *i* from source *j*, given by [*p_ij_* × *m_j_* × *c_j_* / (∑*_j_p_ij_* × *m_j_* × *c_j_*)] × *e_i_*	[4,5,8]
*p_ij_*	Prevalence of subtype *i* from source *j,* given by *π_j_* × *r_ij_*	[1,3,4,16]; this study
*π_j_*	Overall prevalence of *Salmonella* spp. in source *j*	[1,3,4,16,17]
*r_ij_*	Relative frequency of serotype *i* in source *j*	This study
*m_j_*	Amount of source *j* per person per year available on the market (kg for food-animals or number for reptiles)	[4,18,19]
*c_j_*	Probability for foods from source *j* to be eaten raw/undercooked by the population (not applicable for reptiles)	[5,20]
*e_i_*	Frequency of human salmonellosis cases of subtype *i*	Data

We used national surveillance data for all 73,124 laboratory-confirmed human salmonellosis cases reported in the Netherlands during the period from January 1985 to December 2014. Non-typhoid salmonellosis is not a notifiable disease in the Netherlands. However, a national passive surveillance system for *Salmonella* has been in place since 1984, with an estimated 62% coverage of the general population based on a network of diagnostic laboratories that submit *Salmonella* isolates (with accompanying metadata) to the RIVM for further typing [9]. 

Serotyping of all these isolates and further phage typing of the *S*. Enteritidis and *S*. Typhimurium isolates was performed by the national reference laboratory for *Salmonella* at the Dutch National Institute for Public Health and the Environment (RIVM) as described elsewhere [10]. We also used all available *Salmonella* isolates from five putative sources, i.e. pigs (n = 14,395), cattle (n = 11,189), broiler chickens (n = 51,492), table eggs/table egg-laying hens (n = 7,412) and reptiles (n = 2,281) that had been collected during the same period by the Dutch veterinary services (food-producing animals) and private clinics (reptile pets) as part of their routine diagnostic activities and monitoring/surveillance programmes on animals and animal-derived foods at the levels of farm, slaughterhouse and retail (Table 2). Also these isolates were typed at the RIVM within the framework of the national surveillance system for *Salmonella* using the same methods as for the human isolates.

**Table 2:** *Salmonella* subspecies and serotypes in humans (n = 63,718) and animal sources (n = 86,769), the Netherlands, 1985–2014

Subspecies	Serotype	Humans ^a^		Reptiles		Pigs		Cattle		Layers/eggs		Broilers	
n	%	n	%	n	%	n	%	n	%	n	%
*S. enterica* (I)	Typhimurium and its monophasic variant	27,709	43.49	63	2.76	8,984	62.41	4,620	41.29	359	4.84	8,832	17.15
	Enteritidis	18,913	29.68	22	0.96	78	0.54	113	1.01	3,279	44.24	5,200	10.10
	Typhi	402	0.63	0		0		0		0		0	
	Paratyphi A/B/C	487	0.76	1	0.04	14	0.10	4	0.04	82	1.11	4,409	8.56
	Others	16,107	25.28	849	37.22	5,316	36.93	6,450	57.65	3,688	49.80	33,032	64.15
*S. salamae* (II)		24	0.04	276	12.10	1	0.01	0		2	0.03	9	0.02
*S. arizonae* (IIIa)		13	0.02	194	8.51	0		0		1	0.01	2	0.004
*S. diarizonae* (IIIb)		41	0.06	580	25.43	1	0.01	2	0.02	1	0.01	6	0.01
*S. houtenae* (IV)		21	0.03	293	12.85	1	0.01	0		0		2	0.004
*S. bongori/indica* (V/VI)		1	0.00	3	0.13	0		0		0		0	
**Total**		**63,718**		**2,281**		**14,395**		**11,189**		**7,412**		**51,492**	

^a^ Includes only isolates from sporadic, domestic cases.

Of the 73,124 human cases, 5,579 (7.6%) and 1,683 (2.3%) were excluded from the source attribution analysis because they were travel- and outbreak-related, respectively. Another 2,144 cases (2.9%) were excluded because their sero/phage types were not found in any of the considered sources; these cases were then assigned to an unknown source. The model attributed the remaining 63,718 sporadic/domestic cases to the five animal sources. To avoid issues related to sparse data, each year of human cases was attributed based on the subtypes of three years of data for pigs, broilers and layers/eggs (i.e. the same year and the years before and after) and based on all years of reptile data. Interannual trends in the fraction of cases attributed to reptiles were assessed using the Cochran-Armitage test.

## Results

**Figure 1:**
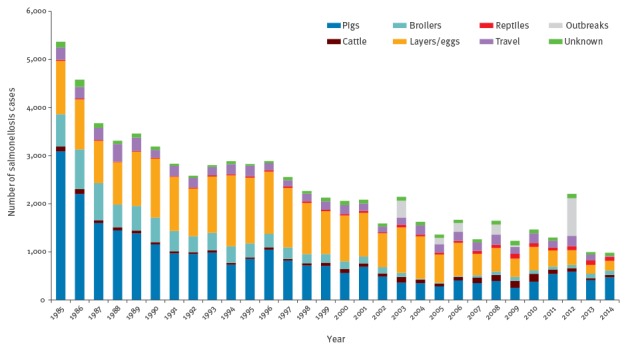
Annual number of reported human salmonellosis cases attributed to different animal sources in the Netherlands, 1985–2014 (n = 73,124)

**Figure 2:**
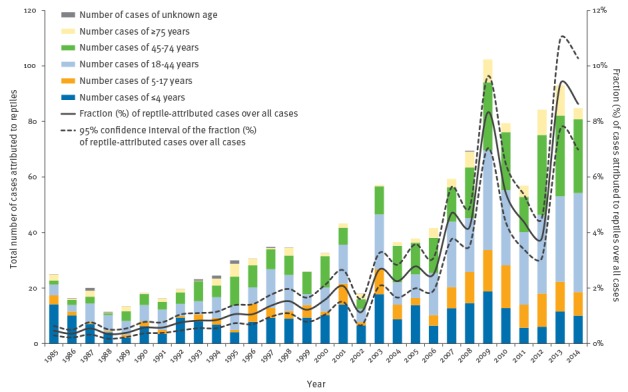
Annual total number of human salmonellosis cases attributed to reptiles, by age group, and estimated fraction of these cases relative to all human salmonellosis cases reported in the Netherlands, 1985–2014 (n = 73,124)

Most reptile isolates (59%) belonged to *S. enterica* subspecies other than subspecies I, particularly to subspecies IIIb (25%). In contrast, the vast majority (> 99%) of human isolates and isolates from food-producing animal belonged to subspecies I (Table 2). In total, 2.0% (95% confidence interval (95% CI): 1.3–2.8) of human cases were attributed to reptiles; attributions to the other sources were as follows: layers/eggs 41.3% (95% CI: 36.0–46.5), pigs 40.9% (95% CI: 36.4–45.5), broilers 12.3% (95% CI: 10.3–14.4) and cattle 3.5% (95% CI: 2.5–4.5). The estimated annual fraction of reptile-associated *Salmonella* infections ranged from a minimum of 0.3% (corresponding to 11 cases) in 1988 to a maximum of 9.3% (93 cases) in 2013 (Figure 1). Although human cases decreased over the years (Figure 1), there was a significant increasing trend (p < 0.0001) in the fraction of reptile-associated *Salmonella* infections (+ 19% on average each year) (Figure 2). Figure 1 also shows the rise and fall of the *S*. Enteritidis epidemic linked to eggs during the 1990s and the growing importance of pigs since the early 2000s (linked to the emergence of *S*. Typhimurium monophasic variant) after a period of evident decline.

Coloured bars, left vertical axis: number of human salmonellosis cases attributed to reptiles; black line, right vertical axis: estimated proportion of human salmonellosis cases attributed to reptiles.

Looking at the age distribution of reptile-associated *Salmonella* infections over the years (Figure 2), the proportion of cases younger than five years relative to the older age groups decreased significantly by 4% annually (p < 0.0001), whereas cases in patients aged 45 to 74 years increased by 20% (p = 0.006) each year.

## Discussion

We showed that despite the observed decline in human salmonellosis cases overall, those associated with reptiles are on the rise and increasingly affecting the adult population. This may be explained by the parallel increase in the trade of live (and often wild-caught) reptiles in the European Union (EU). Although the scale of the illegal market is unknown, 5.9–9.8 million reptiles were (legally) imported into the EU in 2009 alone, a substantial rise from the 1.6 million imported in 2005, which coincided with the ban on wild bird imports placed by the EU in 2005 in response to the H5N1 highly pathogenic avian influenza epidemic in poultry [11]. This lends weight to the hypothesis that the shortage of imported wild birds may have played a role in moving the EU exotic pet market towards reptiles so that prospective and established customers may increasingly have embraced reptiles as pets. This is also mirrored in our attributions, as reptile-associated salmonellosis increased steeply after 2005 (Figure 2).

The observed shift in the age groups at highest risk for reptile-associated salmonellosis may be related to the type of reptiles that are currently kept for companionship. In the past, reptile pets consisted mainly of freshwater aquatic baby turtles like the red-eared slider (*Trachemys scripta elegans*), a popular childhood pet and an important source of salmonellosis for children. As an example, in the United States (US) in the early seventies, pet turtles were responsible for ca 18% of salmonellosis cases among children aged one to nine years [12]. This led to a federal ban in 1975 on the sale of turtles with a shell length less than 10 cm, resulting in a 77% decrease in reptile-associated salmonellosis among children of that age [12]. Although baby turtles have become less popular in the Netherlands since the EU ban on imports of red-eared sliders in 1997 for ethical and environmental reasons, a wider variety of reptile species is currently available on the pet market, and most of these species (mainly lizards and snakes) are clearly meant for adult customers rather than children. This is supported by the increased incidence of venomous (pet) snake bites and other injuries in Europe, extremely rare events until the early 2000s. Further, the importation of these animals has been linked to the changed biodiversity of the European household fauna [13,14]. A resurgence of pet reptiles other than baby turtles is also believed to be responsible for the recent trends in reptile-associated salmonellosis in the US [2].

Our estimate of 2.0% for reptile-associated salmonellosis is in line with previous estimates based on similar source attribution methods [3], but lower than those based on self-reported exposure to reptiles. For instance, in Sweden, 6% of all salmonellosis cases from 1998 to 2000 reported exposure to reptiles [15]. In the US, the population attributable fraction for reptile/amphibian contact was 6% for all sporadic cases from 1996 to 1997, and 11% among those younger than 21 years [2].

## Conclusions

In summary, while human salmonellosis has been decreasing since the 1980s in the Netherlands, we report an increasing trend in reptile-associated salmonellosis and a shift towards adulthood in the age groups at risk, a possible reflection of the increased number and variety of reptiles that are nowadays kept as pets. Although human salmonellosis remains primarily a food-borne disease and the contribution of reptiles is small, our findings call for further attention to the issue of safe reptile ownership in order to target and reinforce current standing recommendations.
